# Control efficacy and groundwater risk of antibiotic resistance genes in semi-arid landfill leachate treatment: seasonal insights and engineering implications

**DOI:** 10.3389/fmicb.2026.1807935

**Published:** 2026-05-15

**Authors:** Ning Chang, Nan Li, Wenhao Li, Jiaying Xue, Yuhong Zheng, Chengzhen Zhao, Shenghu Zhang, Yinqiao Zhang, Guangxuan Yin, Miaoyi Bao, Weitao Shen

**Affiliations:** 1 Department of Engineering, China Pharmaceutical University, Nanjing, China; 2 Department of Gastroenterology, Zhongda Hospital Affiliated to Southeast University, Nanjing, China; 3 Department of Environment Health, Nanjing Municipal Center for Disease Control and Prevention, Nanjing, China; 4 Institute of Agricultural Resources and Environment, Yunnan Academy of Agricultural Sciences, Kunming, China; 5 Nanjing Institute of Environmental Sciences, Ministry of Ecology and Environment, Nanjing, China; 6 Eastern Regional Technology Center of Hazardous Waste Environmental Risk Prevention and Control, Nanjing, China

**Keywords:** antibiotics resistance genes, groundwater contamination risk, leachate, microgenome, season

## Abstract

Landfill leachate is a critical reservoir of antibiotic resistance genes (ARGs) and mobile genetic elements (MGEs), posing prominent risks to groundwater, especially in semi-arid regions. This study focused on the performance of landfill leachate treatment system in Hohhot (Inner Mongolia, semi-arid region), investigating the seasonal variation across three seasons (spring, summer, and autumn), migration characteristics, and control effect of ARGs/MGEs through process optimization-oriented monitoring. Metagenomic sequencing was employed to analyze four key matrices (raw leachate, ultrafiltration effluent, treated leachate, and adjacent groundwater) across three seasons. The treatment system achieved efficient removal of conventional pollutants but failed to eliminate ARGs, MGEs, and antibiotic-resistant bacteria. Instead, it enriched high-risk hosts (e.g., Pseudomonas_E) and transposases (e.g., *tnpA*), exacerbating horizontal gene transfer potential. ARGs abundance showed pronounced peaks in summer and autumn among the sampled seasons. Notably, the resistome profile of treated leachate was highly similar to that of groundwater, indicating incomplete ARG containment and hydrological connectivity between the treatment system and groundwater. A dual-track health-environmental risk framework was applied to the detected ARG subtypes, revealing that overall risk burden was concentrated in a small set of high-priority determinants. The top contributors were dominated by mobility- and co-selection–linked markers (*intI1, tnpA, IS6100, IS26, and qacE△1*) together with clinically relevant resistance genes (*sul1, aacA, and aadA*), underscoring the coupling between resistance functions and genetic mobility in the leachate–groundwater continuum. Collectively, these findings indicate that semi-arid landfill systems can act as both sinks and sources of high-risk resistance determinants, and they highlight the need to integrate ARGs/MGEs-targeted treatment upgrades, seasonally adaptive operational strategies, and risk-based dual-track monitoring into leachate management. This study therefore provides actionable engineering insights for optimizing leachate treatment performance and mitigating cross-media contamination in water-scarce environments.

## 1 Introduction

Antibiotic resistance has been recognized as a critical global health challenge, with profound implications for biowaste management and water resource security. This is largely due to the potential for widespread contamination through industrial activities and waste management practice (Aslam et al., 2024; Bao et al., 2024). The environmental dissemination of ARGs, especially through inadequately treated waste streams, can weaken both ecosystem integrity and public health safeguards (Chen et al., 2025; Chen et al., 2023). Landfill leachate, as a major ARGs reservoir, thus demands integrated treatment-remediation beyond documenting ARGs occurrence, recent omics-based studies highlight that ARGs risks depend on human-associated enrichment, gene mobility, and host pathogenicity—implying not all ARGs pose equal threats. Mobile, human-associated ARGs in pathogenic/opportunistic hosts require higher risk management priority than non-mobile, environment-confined ones. However, this risk-informed perspective has rarely been applied to evaluate or optimize full-scale leachate treatment processes. This study links such a framework with metagenomic evidence from a semi-arid landfill to clarify how treatment processes retain, amplify, or disseminate ARGs across environmental media.

In semi arid regions such as Hohhot in Inner Mongolia, water scarcity further amplifies the environmental and health implications of ARGs spread (Gaur et al., 2024; Huang et al., 2022). Limited precipitation and high evaporation make aquifer systems in these areas especially vulnerable to contamination from leachate leakage or insufficiently treated effluent—threatening the sustainability of local water resource recycling (Ifedinezi et al., 2024). While ARGs occurrence in landfill leachate has been reported, most studies focus on humid climates, leaving a critical knowledge gap regarding ARG behavior and removal efficiency in arid/semi-arid zones. Here, hydrogeological and microbial conditions differ markedly from wetter regions, yet seasonal dynamics of ARGs and treatment performance remain poorly understood from an engineering optimization perspective (Santás-Miguel et al., 2025).

Conventional leachate treatment effectively reduces organic and chemical loads but fails to eliminate ARGs and MGEs—carriers of resistance determinants that persist in treated effluents (Jia et al., 2024). This shortcoming reflects a key engineering gap: current systems merely transfer pollution from solid waste to water rather than achieving complete removal and resource-oriented mitigation. Additionally, the enrichment of high-risk ARG hosts in treated leachate further promotes horizontal gene transfer (HGT), amplifying transmission risks.

To address these underexplored gaps, this study employs metagenomic approaches to track the fate of ARGs, their microbial hosts, and associated MGEs in a full-scale landfill leachate treatment system located in Hohhot – a representative semi-arid region (Pitiot et al., 2025). Four key research questions guide this investigation: (1) How do seasonal variations regulate ARGs prevalence and removal efficiency, and what implications do these patterns hold for seasonal adaptive operation? (2) How do bacterial host communities shift along the treatment train, and can these shifts identify potential ARGs reservoirs? (3) Which indicators effectively reflect elevated HGT potential in final effluents to support process risk screening? (4) What does the resistome similarity between treated leachate and groundwater reveal about contamination pathway blocking strategies? (Anand et al., 2021; Zhang et al., 2022). Specifically, we integrated three complementary analytical layers: taxonomic profiles (hosts) derived from non-redundant (NR) database annotation, resistome composition (ARGs types/subtypes) identified via SARG/ARGs-OAP pipelines, and mobilome features (MGE categories/subtypes) extracted from MobileGeneticElements databases. Downstream network/co-occurrence analyses and source-tracking models were applied to these datasets to interpret ARG dissemination potential across environmental matrices.

By integrating metagenomic evidence with environmental-engineering contexts, this study aims to deliver actionable insights for three core objectives: (1) optimizing treatment system design and operation parameters; (2) enabling safer, integrated control of antibiotic resistance in water-constrained ecosystems; (3) developing ARG transmission barriers for cross-media pollution mitigation. Extending previous research— which often focuses on single treatment units or humid regions—this work introduces three novel technology-application features: (1) combining metagenomic profiling of full-scale leachate treatment with concurrent groundwater monitoring in semi-arid areas, (2) explicitly linking ARGs, MGEs, and resistant hosts to quantify HGT potential along the landfill-leachate-groundwater continuum, (3) applying a dual-track health-environmental risk matrix to rank ARGs subtypes, thereby prioritizing targeted treatment strategies. Collectively, these innovations establish a management-oriented framework for enhancing leachate treatment efficiency and safeguarding groundwater resources in water-scarce regions.

## 2 Materials and methods

### 2.1 Samples collection

**Figure 1:**
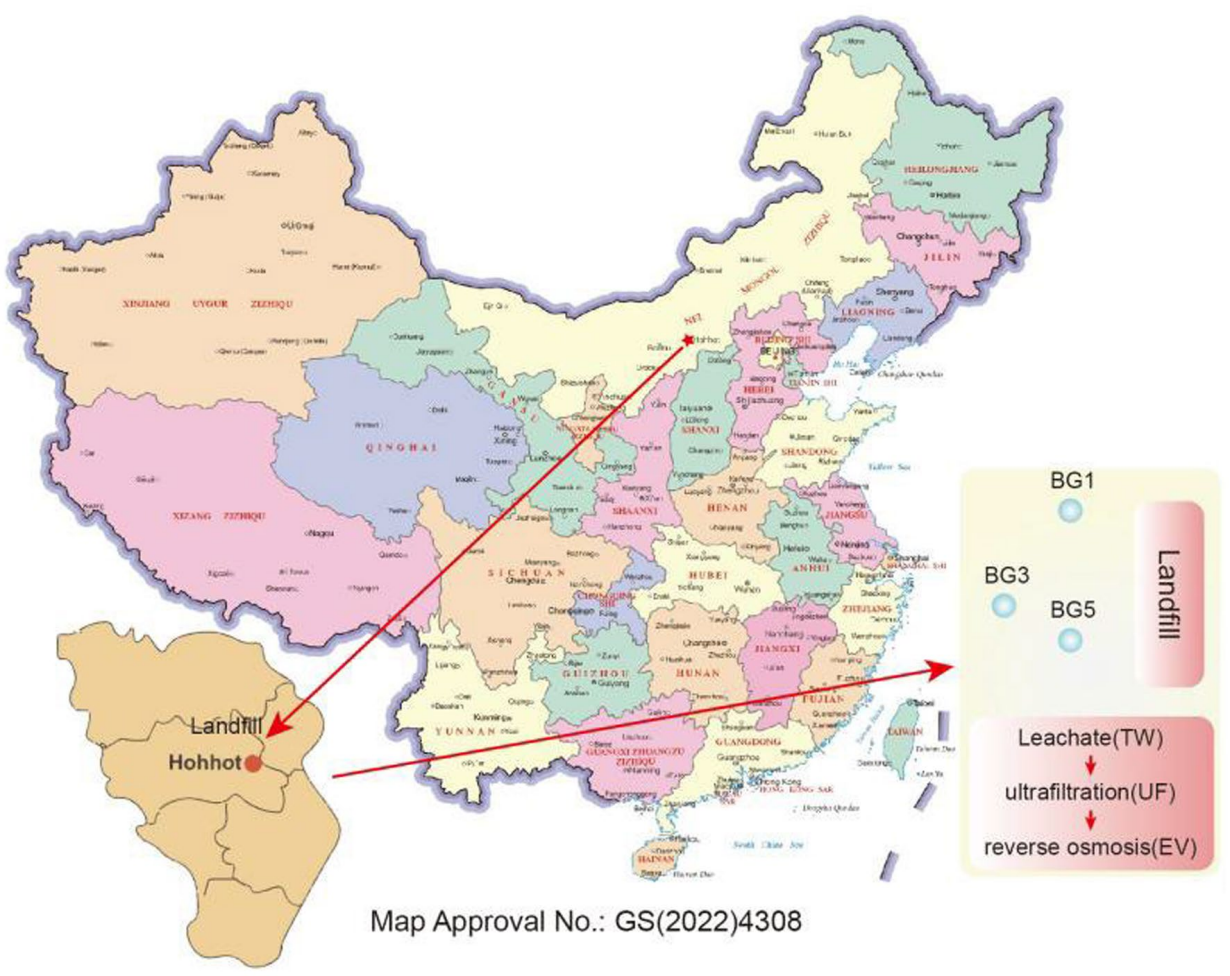
Sample point location diagram.

Our samples were gathered from a typical landfill site and its adjacent groundwater in Hohhot, Inner Mongolia Autonomous Region, China (40.8 °N, 111.7 °E). The leachate treatment plant employs a process of “biological treatment followed by ultrafiltration, nanofiltration (NF), and reverse osmosis (RO),” with a daily treatment capacity of 250 m^3^d^−1^. The leachate treatment process is shown in Figure 1.

Owing to the inaccessibility of certain treatment units and other practical operational constraints, three representative leachate samples were strategically selected to track the control effect and distribution characteristics of ARGs across the core stages of the landfill leachate treatment process chain: raw leachate (IW, collected at the inlet of the ultrafiltration unit), ultrafiltered leachate (UF, collected at the outlet of the ultrafiltration unit), and reverse osmosis treated effluent (EW, collected at the outlet of the RO unit). Specifically, IW represents the resistome profile entering the membrane treatment section after upstream biological treatment, reflecting the intrinsic ARGs/MGEs characteristics of leachate prior to physical barrier treatment; UF permeate was used to characterize the immediate ARGs/MGEs removal performance of ultrafiltration as a key physical barrier in the treatment train; and EW, as the final discharge effluent of the entire treatment process, was employed to assess the overall ARGs/MGEs abatement performance of the downstream advanced polishing steps. Nanofiltration (NF) effluent was not available for collection as the corresponding sampling port was closed for routine maintenance during the sampling campaign. Given the sampling port of NF was closed for maintenance, the differences in ARG profiles between UF permeate and RO effluent (EW) should be interpreted as the combined treatment effects of NF and RO, rather than the independent performance of a single membrane unit. The ultrafiltration unit was configured with 0.02 μm membrane modules and operated at a flux of 60–80 L·m^−2^·h^−1^, whereas the RO unit—equipped with a membrane of nominal pore size 0.1 nm—was run at a stable flux of 10–15 L·m^−2^·h^−1^.

To further investigate the impacts of RO effluent discharge on ARG profiles in groundwater surrounding the landfill and elucidate the environmental migration behavior of leachate-derived ARGs, three groundwater samples (BG1, BG3, and BG5) were collected from monitoring wells in the landfill vicinity. Local hydrogeological surveys revealed that the aquifer in the study area is composed of sandy loam layers with moderate permeability. These groundwater monitoring wells (BG1, BG3, and BG5) are hydraulically connected to the leachate plume, located 100–300 m from the landfill boundary, with the regional groundwater flow direction being from east to west (Figure 1). Sampling was conducted in autumn, spring, and summer. Autumn samples were designated as IW1 (raw leachate), UF1 (ultrafiltration effluent), EW1 (treated leachate), BG11-1 (BG1 groundwater), BG13-1 (BG3 groundwater), and BG15-1 (BG5 groundwater); spring samples as IW2, UF2, EW2, BG21-2, BG23-2, BG25-2; and summer samples as IW3, UF3, EW3, BG31-3, BG33-3, BG35-3.

### 2.2 Analysis of basic physicochemical properties and heavy metals, and antibiotics of samples

In accordance with the established detection protocol, an appropriate volume of raw water sample was procured and subjected to centrifugation at 5000 revolutions per minute for a duration of 10 min, effectively eliminating insoluble impurities. Following this process, the fundamental physical and chemical characteristics of the samples were rigorously evaluated in accordance with both national environmental protection standards and the specifications set forth by the People’s Republic of China. The specific standards adhered to encompass the following: The pH of the samples was meticulously analyzed via the electrode method (HJ 1147-2020); The arsenic content present in the samples was accurately measured utilizing the atomic fluorescence spectrometry method (HJ 694-2014); The concentrations of cadmium, nickel, chromium, copper, manganese, lead, and zinc within the samples were precisely determined by inductively coupled plasma emission spectrometry (HJ 776-2015); The total nitrogen content of the samples was analyzed by gas-phase molecular absorption spectrometry (HJ 199-2005); The total phosphorus content was quantified using ammonium molybdate spectrophotometry (GB 11893-1989).

Regarding the analysis of antibiotic content, an initial volume of 100 mL of untreated leachate was diluted with ultrapure water to a final volume of 1 L. This prepared sample, along with others, underwent analysis for antibiotic content employing the previous methods (Liu et al., 2021).

### 2.3 DNA extraction, library preparation, and sequencing

100 mL of the original leachate was aliquoted and diluted to a final volume of 1 L with sterile water, followed by filtration through a 0.22 μm membrane filter to retain bacterial cells in the sample; this procedure was performed in parallel with all other samples. Total bacterial DNA was extracted from 0.22 μm membrane filters using the DNeasy PowerWater Kit (QIAGEN, Hilden, Germany). Briefly, filters were subjected to bead-beating cell lysis, followed by inhibitor removal, silica-membrane binding, two rounds of washing, and final elution in 100 L of EB buffer. Then, 200 ng of genomic DNA from each sample was fragmented using a Covaris S220 Focused-ultrasonicator (Woburn, MA, USA), and sequencing libraries were constructed with an expected insert size of approximately 450 bp. All libraries were sequenced on an Illumina NovaSeq 6,000 platform with the paired-end 150 bp (PE150) mode. Raw sequencing reads were quality-trimmed using Trimmomatic^1^ to remove adapter contaminants and low-quality reads. After quality control, the remaining reads were aligned to the human reference genome (hg19) using the BWA-MEM algorithm.^2^ Reads free of host genomic contamination and low-quality data were defined as clean reads and subjected to subsequent bioinformatic analysis.

### 2.4 Metagenomic *de novo* assembly, gene prediction, and annotation

The clean sequence reads were organized into contigs employing MegaHit (version 1.1.1), utilizing the parameter “--min-contig-len 500.” Subsequently, the open reading frames (ORFs) of each contig were anticipated using Prodigal (version 2.6.3). All identified ORFs were then consolidated into distinct genes through the application of CD-HIT (version 4.8.1). To ascertain the gene abundance across all samples, Salmon (version 4.8.1) was harnessed to procure the read counts for each individual gene. The gene abundance was derived utilizing the subsequent formula:


Ab(S)=Ab(U)+Ab(M)

$$\mathrm{Ab}(U)=\sum_{i=1}^{M}1/l$$

$$\mathrm{Ab}(M)=\sum_{i=1}^{M}(C_{O}*1)/l$$

$$C_{O}=\frac{\mathrm{Ab}(U)}{\sum_{i=1}^{N}\mathrm{Ab}(U_{i})}$$

Where Ab(S) represents gene abundance, Ab(U) signifies the abundance of single-mapping reads, Ab(M) denotes the abundance of multi-mapping reads, and *l* stands for the length of the gene sequence. The predicted genes were translated into amino acid sequences, which were then compared against the NR, SARG, and MobileGeneticElements database to ascertain the bacterial taxon and MGEs that are present within the sample. To enable cross-sample comparison, we calculated the TPM values for each gene using the following formula:

${\mathrm{TPM}}_{\mathrm{Ab}(S)}=\mathrm{Ab}(S)/\sum \mathrm{Ab}(S)\times 10^{6}$

Annotation of all putative ARGs was performed using the ARGs-OAP (version 3.2). ARGs annotation was performed using 80% amino acid identity and 65% query coverage (E-value <1e-10) against the SARG database. This database employs a hierarchical classification system based on Hidden Markov Models (HMM models), which exhibits higher resolution for ARG variants with high sequence similarity compared to *de novo* clustering at a fixed threshold, thereby effectively reducing misclassification of homologous genes.

Quantitative analysis of ARGs was conducted using TPM as the core metric. For each annotated ARGs locus, the TPM value was calculated via a two-step normalization strategy using the following formulas:

First, compute the gene length-normalized value (Reads Per Kilobase, RPK):


$${\mathrm{RPK}}_{\text{ARGs}}=\frac{\text{Number of reads mapped to the target ARGs}}{\text{Length of the target ARGs}(\mathrm{kb})}$$

Subsequently, perform sample-level normalization to obtain TPM:


$${\mathrm{TPM}}_{\text{ARGs}}=\frac{\mathrm{RPK}\;\text{of the target ARGs}}{\mathrm{Sum}\;\text{of}\;\mathrm{RPK}\;\text{values of}\;\mathrm{all}\;\text{genes in the sample}}\times 10^{6}$$

The analytical pipeline established in this study, combining “ARGs-OAP annotation+TPM quantification,” specifically addresses two key limitations of traditional clustering strategies:

*Preservation of functional homology*: Database-based annotation enables the aggregation of functionally equivalent ARGs variants at the gene family level, preventing the amplification signals or mobility potential of resistance genes from being fragmented across multiple functionally unrelated clusters and ensuring the integrity of biological information.

*Improved quantification accuracy*: It allows for precise assessment of the relative total abundance of various resistance determinants, truly reflecting the prevalence and expression potential of ARGs in the microbial community. Notably, TPM’s inherent normalization for sample composition differences makes it particularly suitable for the abundance analysis of mobile resistance genes and multi-copy resistance genes, as well as cross-sample comparative studies.

*Terminology definition for abundance quantification*: For the convenience of cross-sample comparison and result interpretation, three key terms related to abundance quantification are clearly defined as follows: (1) Read counts: The raw number of sequencing reads mapped to each predicted gene, without any normalization; (2) Gene abundance: The length-corrected gene abundance calculated by the formula in this section, which eliminates the bias caused by differences in gene sequence length; (3) Relative abundance: The TPM-normalized abundance (calculated by the formula in this section) of ARGs or bacterial taxa, which is the core metric for cross-sample comparative analysis in this study, as it normalizes the total gene abundance of each sample to 10^6^.

### 2.5 Health and environmental analysis and assessment

This study builds on the three dimensional framework proposed by previously research (Zhang et al., 2021). That framework evaluates the health relevance of ARGs from three aspects. These aspects are human associated enrichment, gene mobility and host pathogenicity. We first used these criteria to classify each detected ARGs into four health risk ranks from Rank I to Rank IV. For each ARGs we considered its enrichment in human related environments, its association with mobile genetic elements and its detection in ESKAPE pathogens. We then further divided Rank I ARGs families. Families already listed by the World Health Organization were separated from Rank I families that are not yet listed. Based on this extension we constructed a five level health risk track that we labeled H1 to H5. The relevant parameters for risk calculation are provided in Supplementary material S1. In this study, the H1-H5/E1-E5 matrix is applied as an exploratory, data-driven screening tool for within-system prioritization. The scoring thresholds and leakage weights are derived from the present dataset, and external validation across independent landfill leachate treatment systems has not yet been performed; this limitation will be discussed in Section 4.6.

For the environmental track we defined indicators that describe ARGs behavior in the leachate–groundwater system. First, for each subtype we calculated its mean relative abundance A across all leachate and groundwater samples. This metric reflects the overall prevalence of an ARG within the system and is used as a relative indicator for internal comparison. Second, we assigned a semi quantitative leakage factor L to each subtype. ARGs detected in both treated leachate and groundwater, with similar resistome profiles, received L = 1.0. ARGs detected in treated leachate and occasionally in groundwater received L = 0.5. ARGs confined to leachate samples received L = 0.1. This factor represents relative leakage likelihood rather than a mechanistic probability. We then calculated an environmental score E_score = A × L for each subtype and divided all values into five equal sized groups based on dataset specific quintiles. These groups were labeled E1 to E5 from lowest to highest environmental score.

**Figure 2:**
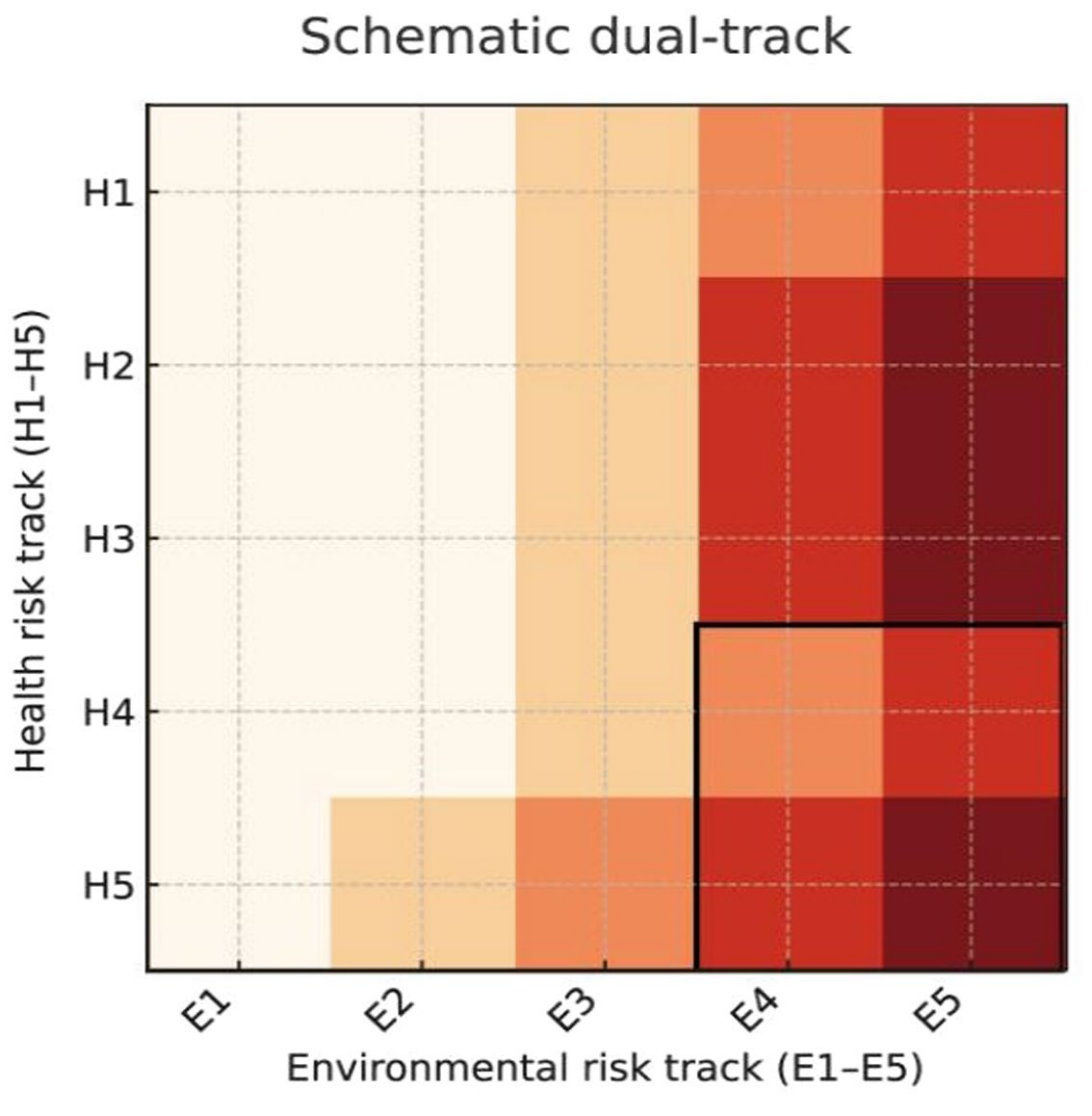
Schematic diagram of the dual assessment system for health and environmental risks.

Finally, we combined the health track and the environmental track in a dual track risk matrix. The matrix has health risk levels H1 to H5 on the y axis and environmental risk levels E1 to E5 on the x axis. We first plotted a schematic matrix to visualize the structure of the framework. Colors gradually intensify from the upper left to the lower right corner and show increasing combined risk (Figure 2). In this visual representation the cells in the H4 to H5 by E4 to E5 block in the lower right define a high–high region, which we used to identify ARG subtypes with both elevated health relevance and strong environmental persistence. For descriptive purposes we also calculated a simple combined risk index for each subtype. We coded H1 to H5 as 1 to 5 and E1 to E5 as 1 to 5 and multiplied these two scores.

### 2.6 Statistical analysis

Co-occurrence networks were structured utilizing Spearman’s correlation analysis and visualized through Cytoscape (version 3.8.0). The SourceTracker was employed to quantify the relative contributions of ARGs in treated leachate to groundwater. Statistical analysis was performed and figures were prepared using the Biozeron Cloud Platform,^3^ SPSS software (version 20.0; SPSS Inc., Chicago, IL, USA), and Prism software (GraphPad Software Inc., La Jolla, CA, USA).

## 3 Results

### 3.1 Analysis of basic physicochemical properties, heavy metals, and antibiotic contents

The comprehensive analysis of the physicochemical properties and heavy metal concentrations in leachate revealed that the leachate treatment process effectively mitigates the levels of total nitrogen, total phosphorus, COD, and various heavy metals in raw leachate. The removal rates of total nitrogen, total phosphorus, COD, and heavy metals exceeded 90%, with the exception of Mn, whose removal rate was approximately 65% in the spring group (IW2 to EW2) (Supplementary Table S1). This indicates that the current treatment system significantly improves leachate quality and reduces conventional pollutant loads. Seasonal variations were observed in the raw leachate, with higher pH and total nitrogen concentrations recorded in spring. In autumn groundwater samples, pH was relatively high, and Cr and Ni were below detection limits across all groundwater sites. Otherwise, the analysis results of antibiotics indicate that the leachate treatment process is capable of effectively removing antibiotic residues from the leachate.

### 3.2 Basic data of metagenomic sequencing

This study analyzed a total of 54 samples, including 27 groundwater samples and 27 samples from raw leachate, ultrafiltration effluent, and treated leachate. All samples were subjected to high-throughput sequencing on the Illumina platform, yielding high-quality data with Q30 ≥ 81.85%, sequencing volumes ranging from 6.03 to 17.47 Gb per sample, and low error rates, supporting reliable downstream metagenomic analysis. After quality filtering and adapter trimming using Trimmomatic, clean reads were *de novo* assembled with MEGAHIT, a tool widely recognized for efficient metagenomic assembly. In total, 6,349,619 contigs were generated from groundwater samples (average 235,171 contigs per sample), and 5,405,397 contigs were obtained from raw leachate, ultrafiltration effluent, and treated leachate samples (average 200,200 contigs per sample). Across all samples, the number of contigs ranged from 111,746 to 616,085, with total assembled lengths of 90.76–805.76 Mb, N50 values of 1,180–3,839 bp, and maximum contig lengths up to 1.55 Mb, demonstrating satisfactory assembly continuity for subsequent taxonomic and functional analysis. Specific sequencing quality data for each sample are provided in Supplementary material S3.

Using META Prodigal, optimized for metagenomic gene prediction, we identified 13,265,421 Open Reading Frames (ORFs) in groundwater samples (average 491,665 ORFs/sample) and 11,844,057 ORFs in the combined leachate-related samples (raw leachate, ultrafiltration effluent, and leachate samples, mean = 438,669 ORFs/sample). To generate a high-quality, non-redundant gene set, we employed CD-HIT clustering software with stringent parameters (95% sequence identity and 90% coverage). The longest sequence in each cluster was retained as the representative to minimize potential chimeras. This comprehensive gene catalog provided the foundation for subsequent taxonomic and functional analyses.

### 3.3 ARG profiles in landfill leachate treatment and adjacent groundwater

**Figure 3:**
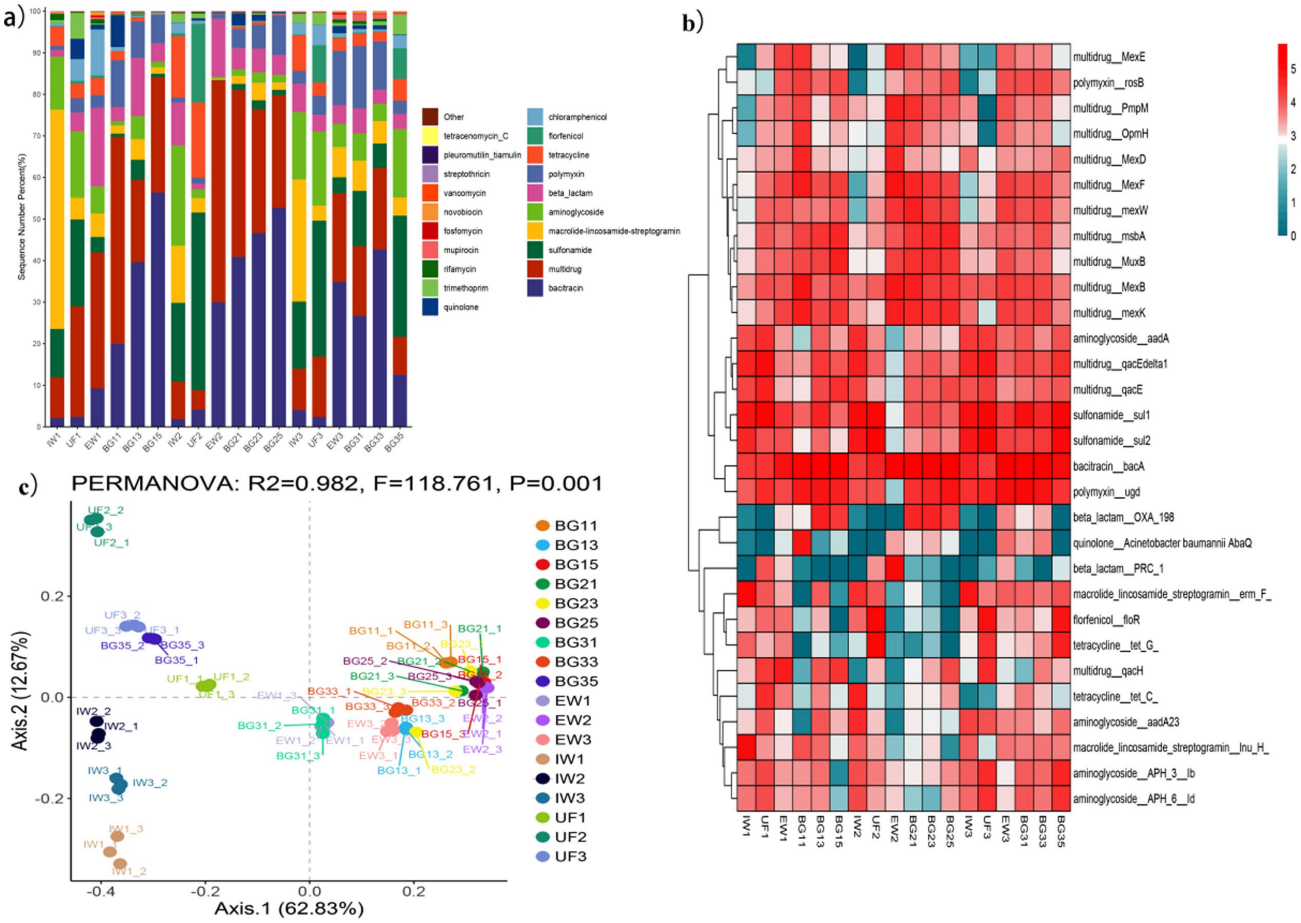
Compositional characteristic of ARGs in leachate treatment processes and groundwaters. **(a)** ARG type composition across different samples; **(b)** ARG subtype composition across different samples; **(c)** PCoA analysis of ARGs across different samples.

Comprehensive analysis utilizing the ARGs-OAP revealed 26 ARG types, with notable variations in their distribution patterns across temporal sampling periods. Analysis of raw leachate samples collected during autumn, spring, and summer identified 22 (IW1), 20 (IW2), and 21 (IW3) ARG types, respectively. Macrolide-lincosamide-streptogramin ARGs (MLS-ARGs) were predominant in IW1 and IW3, while aminoglycoside-ARGs showed the highest relative abundance in IW2. Analysis of ultrafiltration (UF) effluent identified 23, 18 and 18 ARG types in UF1 (autumn), UF2 (spring) and UF3 (summer), respectively; multi-ARGs (multidrug resistance genes: genes conferring microbial resistance to two or more distinct antibiotic classes) dominated in UF1, with sulfonamide-ARGs predominant in UF2 and UF3. The final treated leachate contained 23, 16 and 21 types of ARGs in EW1, EW2 and EW3, respectively; multi-ARGs dominated in EW1 and EW2, while bacitracin resistance genes were the most abundant in EW3. Notably, this treatment process demonstrated limited efficacy in removing ARGs, with treated leachate still exhibiting a relatively high detection rate of ARGs compared to untreated leachate (Figure 3a).

Patiotemporal analysis of groundwater near the landfill revealed distinct heterogeneity in ARG profiles. The number of ARG types detected was 22 (BG11), 18 (BG21), and 22 (BG31) at well BG1; 22 (BG13), 20 (BG23), and 21 (BG33) at well BG3; and 19 (BG15), 18 (BG25), and 18 (BG35) at well BG5 during autumn, spring, and summer, respectively. Bacitracin ARGs predominated at most groundwater sites, except in BG11 (autumn) and BG35 (summer), where multidrug ARGs and sulfonamide ARGs were most prevalent, respectively. Overall, 1,002 ARG subtypes across 26 antibiotic resistance categories were detected (Figure 3a).

In autumn, the number of detected ARG subtypes increased gradually exhibited a gradual increase throughout the treatment process, with 334 subtypes in IW1, 407 in UF1 and 480 in EW1. Correspondingly, *erm(F)*, *sul1* and *bacA* were the subtypes with the highest relative abundance in IW1, UF1 and EW1, respectively. In spring, the number of ARG subtypes decreased progressively: 342 in IW2, 293 in UF2, and 180 in EW2. The dominant subtypes were *sul1*, *sul2*, and bacA in the three sequential stages. For the summer group (IW3/UF3/EW3), the number of ARG subtypes first decreased then increased: 311 in IW3, 237 in UF3, and 381 in EW3. The dominant subtypes followed the same pattern as spring (*sul1* in IW3, *sul2* in UF3, and *bacA* in EW3). Clear seasonal shifts were observed, and treated effluent generally showed higher ARG abundance than raw leachate, especially in spring (Figure 3b).

In groundwater, the number of ARG subtypes at BG5 was significantly lower than at BG1 and BG3 across autumn (303 in BG11, 388 in BG13, 264 in BG15), spring (267 in BG21, 269 in BG23, 187 in BG25), and summer (385 in BG31, 358 in BG33, 240 in BG35), indicating lower impact from leachate-derived ARGs compared with wells closer to the landfill (Figure 3b).

In the principal coordinate analysis (PCoA), all samples were generally clustered into three groups: raw leachate, ultrafiltration effluent, and a combined cluster of treated effluent and most groundwater samples. Notably, ARG profiles of groundwater showed high similarity to treated leachate (except summer BG35), implying a close genetic association between leachate and groundwater resistomes (Figure 3c).

### 3.4 Analysis of the removal effect of ARGs in leachate by the leachate treatment process

**Figure 4:**
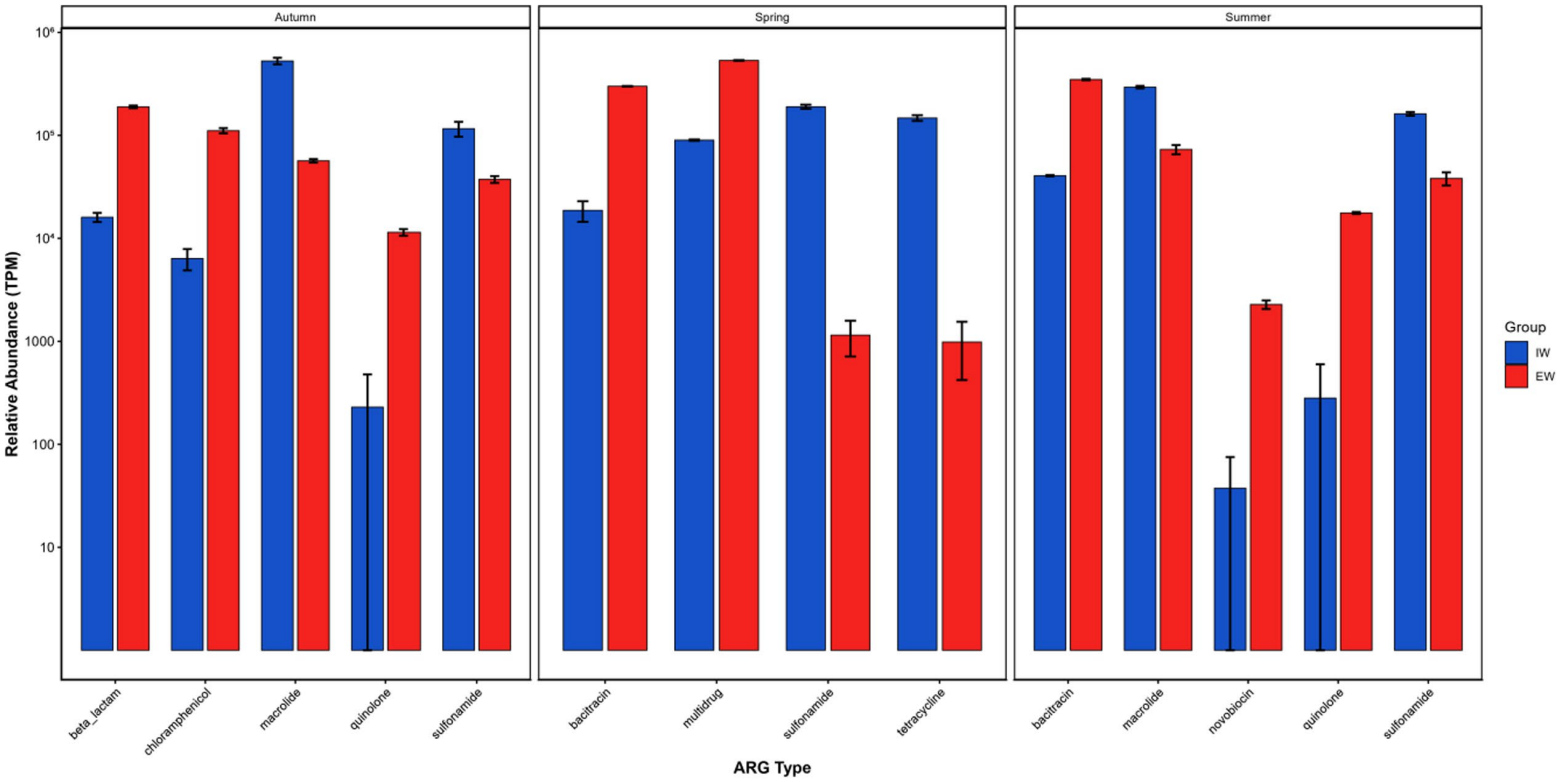
The impact of leachate treatment processes under different seasons on the relative abundance of ARGs in leachate.

Statistical analysis was performed using the Mann–Whitney U test to compare ARG control efficiency and seasonal dynamics during leachate treatment. Given the limited biological replicates per group, differential ARGs were identified based on biological effect size using a threshold of |log_2_FC| > 1 (fold change > 2) (Figure 4).

Seasonal comparison of ARGs relative abundances between EW and IW groups revealed distinct seasonal patterns. In autumn, 8 ARGs showed strong differential trends between EW1 and IW1. Of these, 4 ARGs (dominated by quinolone- and chloramphenicol- ARGs, with 50-fold and 17-fold elevations, respectively) were markedly enriched, while 4 ARGs (mainly MLS- and sulfonamide- ARGs, with 9-fold and 3-fold reductions, respectively) were notably depleted. In spring, 12 ARGs exhibited strong differential trends between EW2 and IW2: 4 ARGs (primarily bacitracin- and multi-ARGs, with 16-fold and 6-fold enriched) were increased, whereas 8 ARGs (predominantly sulfonamide- and tetracycline- ARGs, with 167-fold and 143-fold depleted) were decreased. In summer, 12 ARGs displayed distinct abundance trends between EW3 and IW3, with 7 ARGs (mainly quinolone- and novobiocin ARGs, showing 63-fold and 61-fold surges) markedly elevated, and 5 ARGs (dominated by sulfonamide- and MLS- ARGs, displaying 4-fold and 4-fold reductions) notably reduced.

Collectively, these findings demonstrate that seasonal variation acts as a prominent driver shaping ARG profiles between the two groups, characterized by seasonal enrichment of specific ARG classes (e.g., quinolone, bacitracin, novobiocin) and marked depletion of others (notably sulfonamide, tetracycline). These observations underscore the critical role of seasonal variation in shaping the distribution and abundance of ARGs.

### 3.5 Co-linearity network of ARGs during leachate treatment process

Co-occurrence patterns were established through Pearson correlation analysis (*p* < 0.05, |r| > 0.7) thereby elucidating the core ARGs and their potential interactive relationships throughout the leachate treatment process (Supplementary Figures S1a–c).

In autumn samples, *erm(T), mexW, erm(B), mexF, and smeE* were identified as the dominant hub genes in the co-occurrence network. By spring, the network topology had undergone significant reconfiguration, with *mexJ*, *adeF*, *catB11*, *aadA22*, and *dfrA1* emerging as central nodes. Temporal evolution was further observed in summer samples, where the co-occurrence network architecture revealed *aadS,erm(35), pmpM, muxB, and erm(B)* as putative hub nodes. It should be noted that these network patterns only indicate potential ARG co-occurrence, rather than providing definitive evidence for HGT among them.

Notably, the topological characteristics of these seasonal co-occurrence networks exhibited distinct temporal variations. The autumn and summer networks were dominated by positive correlations, implying synergistic interactions among ARGs during these two seasons. In contrast, the spring network displayed a predominance of negative correlations, suggesting the prevalence of antagonistic ARG interactions in spring. Collectively, these temporal shifts in network topology demonstrate that ARG interactive patterns during leachate treatment are seasonally dependent: positive correlations are the primary interaction type in autumn and summer, while negative correlations prevail—in spring.

### 3.6 Characteristic analysis of ARGs migration in leachate to groundwater

PCoA revealed substantial compositional similarities between ARG profiles in treated leachate and groundwater samples, suggesting potential dissemination of ARGs from leachate to groundwater and subsequent shifts in the groundwater resistome (Huang et al., 2022). To further investigate this relationship, correlation analyses were performed.

Linear regression showed significant temporal and spatial patterns between relative abundances of ARGs in treated leachate and groundwater (Supplementary Figures S2a–i). During autumn, linear correlations were observed between the relative abundance of ARGs in treated leachate and groundwater samples (*p* < 0.001), though the correlation strength varied by sampling site: BG11, BG13, and BG15 samples exhibited R^2^ of 0.67, 0.39, and 0.28, respectively. The strongest linear correlations were detected in spring (*p* < 0.001), with R^2^ values reaching 0.89 for BG21, 0.73 for BG23, and 0.64 for BG25. In summer, significant linear correlations were identified for BG31 (R^2^ = 0.89, *p* < 0.001), BG33 (R^2^ = 0.97, *p* < 0.001), and BG35 (R^2^ = 0.18, *p* < 0.001). These results indicate stable linear associations across seasons, although explanatory power differed by site.

To further evaluate the contribution of treated leachate to the groundwater resistome, SourceTracker analysis was used to estimate the proportional contributions of leachate-derived ARGs to groundwater (Supplementary Figures S3a–c). This analysis showed high concordance between the resistomes of groundwater and treated leachate, supporting potential ARGs transfer from leachate to groundwater. In autumn, treated leachate contributed approximately 80, 87, and 92% to the ARG profiles of BG11, BG13, and BG15, respectively. In spring, contributions remained high at 90% (BG21), 84% (BG23), and 91% (BG25). In summer, contributions were 82% (BG31), 93% (BG33), and 36% (BG35), respectively.

Collectively, these findings suggest that ARGs dissemination from treated leachate to groundwater may occur and exhibits spatial variability. However, these findings are based on observational bioinformatic evidence and require further experimental validation to confirm direct transmission, underlying mechanisms, and associated ecological risks.

### 3.7 Characteristics of bacterial occurrence and composition in leachate treatment processes and groundwater

Employing the NR library to analyze bacterial composition in leachate treatment processes, this study examined the impact of these processes and sampling times on the microbial structure of leachate. Results revealed that *Pseudomonadota* and *Bacteroidota* were the dominant bacterial phyla in raw leachate, irrespective of seasonal changes. Post-ultrafiltration treatment, *Pseudomonadota* and *Bacteroidota* persisted as the primary bacteria in the UF effluent during autumn and summer. However, in spring, *Planctomycetota* and *Pseudomonadota* became the prevalent bacteria in the UF effluent. Following further treatment, *Pseudomonadota* and *Bacteroidota* emerged as the leading bacteria in the treated leachate, irrespective of seasonal changes. In summary, at the phylum level, *Pseudomonadota* was the most abundant bacterium throughout the entire leachate treatment process.

**Figure 5:**
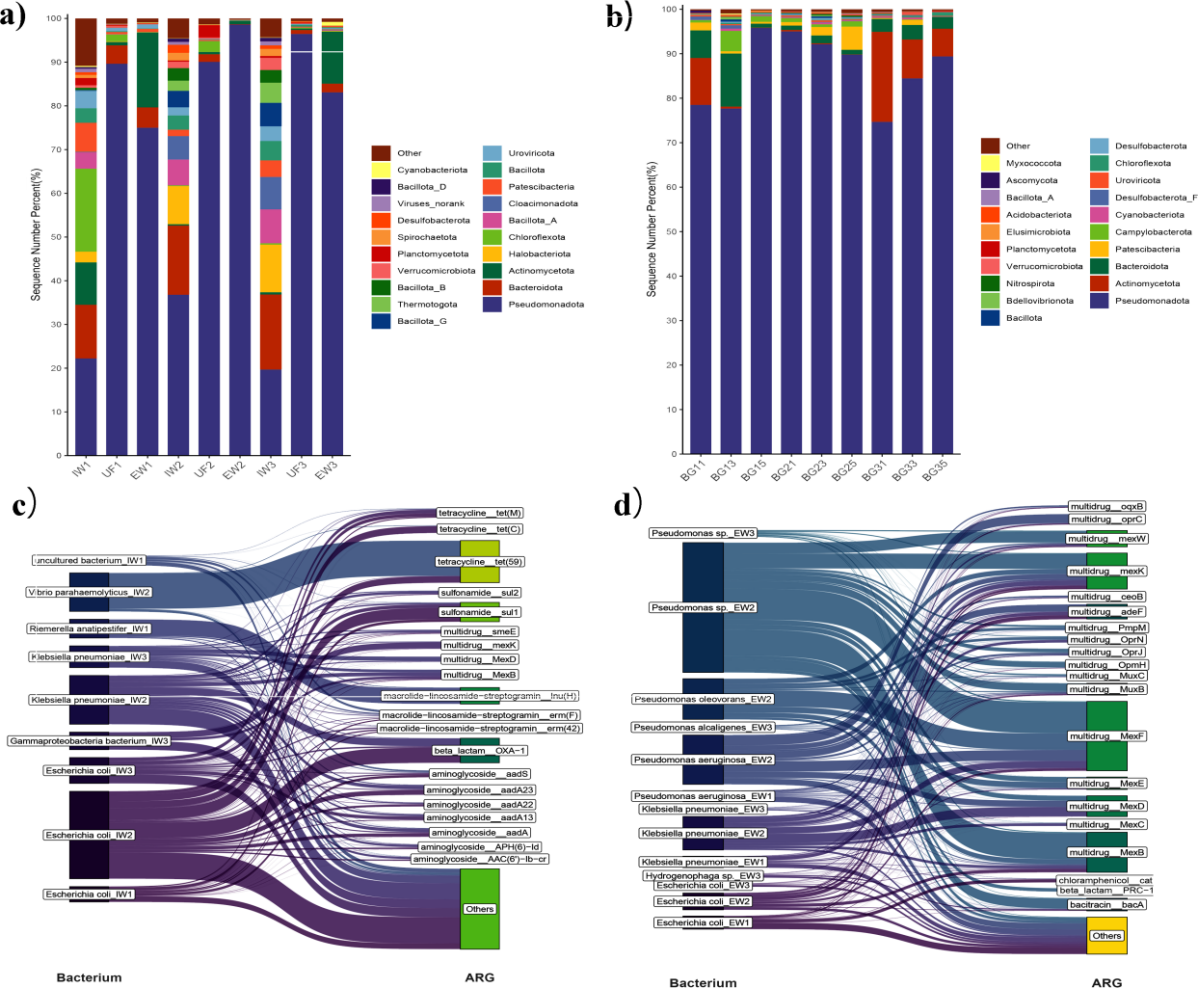
Composition of bacteria and antibiotics-resistant bacteria in the sample. **(a)** Stands for the bacterial composition in leachate treatment processes. **(b)** Stands for the bacterial composition in groundwater. **(c)** The situation of main ARBs in the raw leachate. **(d)** The situation of main ARBs in the treated leachate.

Furthermore, the study analyzed the effect of the leachate treatment process on the bacterial abundance in raw leachate. Findings indicated that in autumn, the relative abundance of *Pseudomonadota* in EW1 increased by four-fold, while that of *Bacteroidota* decreased two-fold, compared to IW1; In spring, the relative abundance of *Pseudomonadota* in the EW rose three-fold, and that of *Bacteroidota* diminished 144-fold, compared to IW2; In summer, the relative abundance of *Pseudomonadota* in the EW escalated five-fold, and that of *Bacteroidota* dropped eight-fold, composed to IW3. In summary, after treatment, the proportion of *Pseudomonadota*/*Bacteroidota* in the leachate significantly increased. Figures 5a,b displays the microbial composition of the phylum-level in leachate.

As for groundwater, the results found that the main bacterial groups in the groundwater samples are similar. In autumn, the main bacteria in the groundwater samples were *Pseudomonadota* and *Bacteroidota*. Additionally, *Actinomycetota* in BG1 and Campylobacter in BG3 and BG5 also had a relatively high abundance. In the groundwater samples collected in spring, *Pseudomonadota* emerged as the predominant bacterium, with *Bacteroidota* and Campylobacter also exhibiting a high relative abundance in BG1. Furthermore, BG3 displayed a significant relative abundance of both *Bacteroidota* and *Patescibacteria*, while BG5 had a high relative abundance of *Patescibacteria* and Campylobacter. By summer, the groundwater samples revealed that the principal bacteria present were *Pseudomonadota*, *Actinomycetota*, and *Bacteroidota*.

### 3.8 Characteristics of antibiotic-resistant bacteria in leachate treatment process and groundwater

We integrated the NR and SARG databases to investigate the characteristics of ARBs in leachate treatment processes and groundwater. The analysis focused on the influence of leachate treatment on ARB composition and the seasonal variations in ARB, as determined by sampling times.

A total of 2,276 potential ARBs were identified in IW, which predominantly harbored MLS-ARGs, sulfonamide-ARGs, and multi-ARGs. For the sample-specific distribution, 1,408, 1,288, and 1,079 potential ARBs were detected in IW1, IW2, and IW3, respectively. *Riemerella anatipestifer* and *Escherichia coli* were the dominant ARB taxa in IW1, carrying 15 and 100 distinct ARG types each. In IW2, *E. coli* and *Klebsiella pneumoniae* exhibited the highest ARGs diversity, with 131 and 108 distinct ARG types identified, respectively; in another subset of IW2, *ugd*, *mexB* and *smeE* were associated with a relatively large bacterial host range, corresponding to 165, 79 and 65 host species. Notably, ugd, *mexB* and *bacA* were also found to have a broad bacterial host distribution in IW2, with 199, 84 and 79 host species detected separately. In IW3, the dominant ARBs were still *E. coli* and *K. pneumoniae*, which contained 118 and 86 distinct ARG types each. Additionally, *bacA*, *mexB* and *smeE* in IW1 were hosted by a large number of bacterial taxa, with 112, 111 and 108 corresponding host species, respectively.

The number of potential ARBs increased significantly to 3,229 in EW, with 2038, 900, and 2,174 ARBs detected in EW1, EW2, and EW3, respectively. *E. coli* and *K. pneumoniae* were the dominant ARBs in EW1, harboring 166 and 132 distinct ARG types each. In EW2, *K. pneumoniae* and *Pseudomonas aeruginosa* showed the highest ARG diversity with an equal number of 45 distinct ARG types for each. In EW3, *E. coli* and *K. pneumoniae* contained 96 and 83 distinct ARG types, respectively. Moreover, *mexF* and *mexB* were associated with the largest bacterial host range in the treated leachate, with more than 180 bacterial host species detected across EW1, EW2, and EW3. This broad host range indicated that these ARGs were widely distributed in the microbial community of treated leachate, despite their differences in clinical relevance. Collectively, our results demonstrated that leachate treatment could significantly alter the composition and diversity of ARG host taxa in leachate. However, the composition of ARBs in the treated leachate was also associated with seasonal variations. The distribution of ARBs in raw and treated leachate are presented in Figures 5c,d.

Analysis of groundwater samples (BG1, BG3, and BG5) revealed a considerable diversity of ARBs. Specifically, 2031, 2049, and 1,230 ARB species were identified in autumn-collected BG1, BG3, and BG5 samples, respectively. *E. coli*, *K. pneumoniae*, and *P. aeruginosa* were the dominant ARBs in BG1, BG3, and BG5, with each harboring more than 60 distinct types of ARGs. Further analysis demonstrated that the dominant ARB taxa in groundwater samples collected in spring and summer were consistent with those detected in autumn. Multi-ARGs were the most prevalent ARG type across all groundwater samples. Additionally, *bacA* exhibited the highest bacterial host diversity among all ARGs in groundwater samples; with the exception of BG1 in autumn, over 200 bacterial host species were identified for this gene in each sample. This pattern indicates that *bacA* is a widespread environmental resistance determinant in the aquifer microbiome, although its value as a specific clinical risk marker is limited.

### 3.9 Occurrence characteristics of MGEs

**Figure 6:**
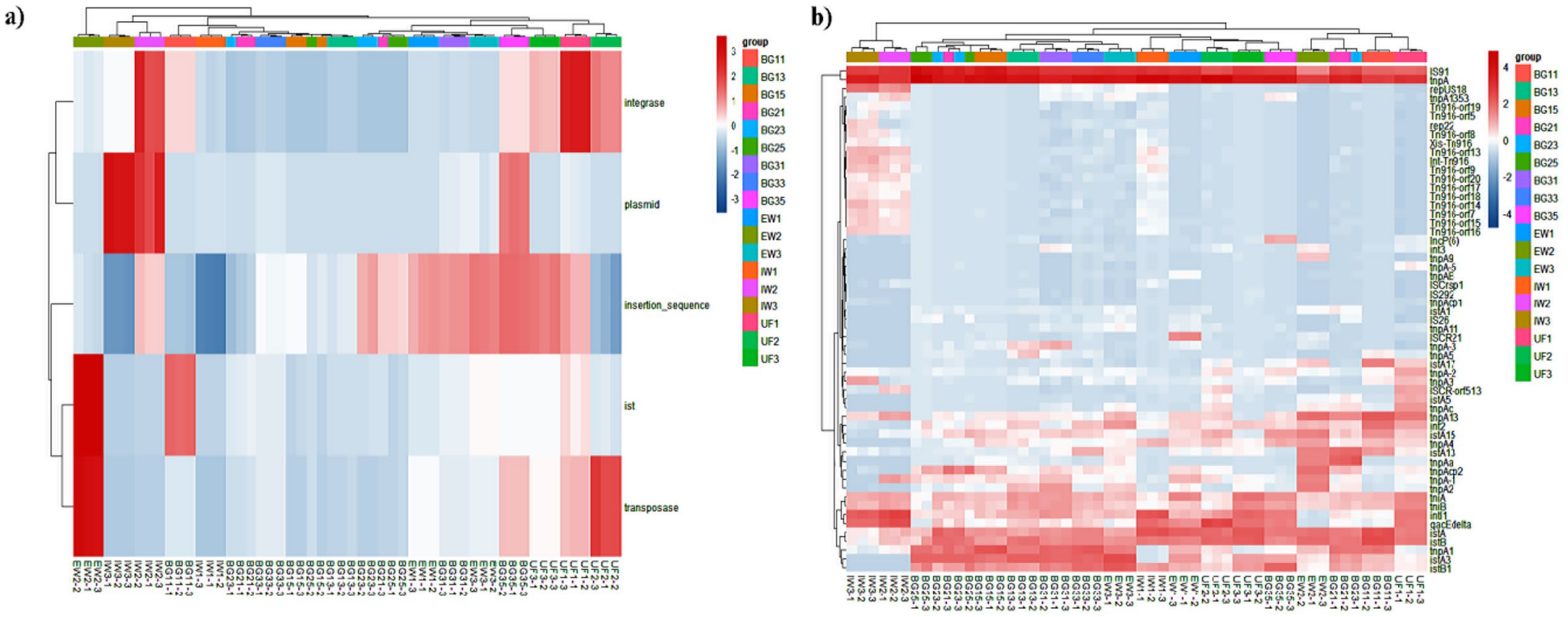
Leachate treatment processes (raw leachate, ultrafiltered leachate, and treated leachate) and the relative abundance of MGEs **(a)** and their subtypes **(b)** in groundwater.

A total of 57 MGE subtypes were annotated in leachate treatment units (IW, UF, EW) and adjacent groundwater samples (BG1, BG3, BG5) collected in spring, summer and autumn, with transposases, insertion sequences, and integrases as the dominant categories and plasmids exhibiting the lowest relative abundance in all samples (Figures 6a,b). *tnpA*, *IS91*, *istA* and *istB* were the core dominant subtypes in all matrices, with their relative abundances markedly exceeding those of other MGEs. The transposase-encoding gene *tnpA* maintained the highest abundance in all sample types and seasons, followed by the insertion sequence *IS91* and integrase gene *intI1*. Spatially, groundwater samples harbored the same core dominant MGEs as leachate samples, with BG1 consistently showing the highest MGE abundances (e.g., average *tnpA* abundance of 569.62 in autumn BG11) and BG5 showing the lowest (e.g., average *tnpA* abundance of 302.53 in autumn BG15) across seasons, presenting a clear spatial gradient consistent with the impact of leachate infiltration. Temporally, the MGE detection rate in raw leachate was 84% in autumn (IW1), 67% in spring (IW2), and 68% in summer (IW3). The primary MGEs in each season were *tnpA*, *IS91*, and *istA* (IW1), *IS91*, *intI1*, and *tnpA* (IW2), and *IS91*, *intI1*, and *tnpA* (IW3), respectively. Groundwater samples were predominated by *tnpA*, *istB*, and *istA* in autumn, and by *IS91* and *tnpA* in spring and summer across the sampling periods.

### 3.10 Variation characteristics of MGEs

Statistical analysis was performed using the Mann–Whitney U test to compare MGE dynamics during leachate treatment. Given the limited biological replicates per group, differential MGEs were identified based on biological effect size using a threshold of |log2FC| > 1 (fold change > 2).

MGEs exhibited distinct temporal variation patterns during the leachate treatment process, with a general trend of decreased detection rate but elevated relative abundance of dominant MGEs from IW to EW, expect summer, and marked seasonal differences in the fluctuation extent of different MGE categories.

In autumn, the MGE detection rate decreased from 84% (IW1) to 67% (EW1); the relative abundance of insertion sequences, ist-type elements, and transposase genes showed fold changes > 2 in EW1 compared to IW1, with core subtypes *tnpA*, *IS91*, and *istA* increasing by 8-fold (152.64 to 1265.75), 7-fold (32.88 to 248.68), and 4-fold (16.59 to 64.13) in average relative abundance, respectively.

In spring, the MGE detection rate slightly decreased from 67% (IW2) to 65% (EW2); integrase and transposase genes increased by 76.2-fold and 16.9-fold, with *tnpA* showing a 17.4-fold increase (245.79 to 4238.42 in average relative abundance), while *IS91* and *intI1* showed reduced relative abundance, and subtypes including *tnpA2*, *tnpAcp2*, and *istB* showed increased abundance in EW2.

In summer, the MGE detection rate increased sharply from 68% (IW3) to 95% (EW3), differing from the other two seasons; ist-type elements, transposase genes, and insertion sequences all showed increased abundance in EW3, with *IS91* and *tnpA* increasing by 4.4-fold (230.36 to 305.85 for *IS91*) and 4.6-fold, respectively, while *intI1* showed a decreasing trend consistent with that in spring.

Notably, the transposase gene *tnpA*, which had high initial abundance in raw leachate, showed consistent increases after membrane treatment across all seasons, and also remained at high relative abundance in groundwater samples throughout the study period. To better understand subtype-level patterns and their potential links to ARG risk, *tnpA* encodes a transposase that mediates the transposition of insertion sequences and transposons, facilitating the mobilization of ARG-carrying DNA segments. The *intI1* gene encodes the integrase of class 1 integrons, which promotes the capture and recombination of gene cassettes frequently associated with ARGs; thus, its presence indicates the potential for integron-mediated ARG dissemination.

### 3.11 Analysis of host for mobile genetic elements

Integrate the annotation results of MGEs with the bacterial taxonomic annotation results from the NR database to further identify the hosts of MGEs in leachate treatment processes, and to evaluate the potential risk of horizontal gene transfer mediated by MGEs for ARGs. Upon conducting a meta-analysis, we determined that 82% (1,411/1720) of the contigs related to MGEs are associated with specific bacterial hosts (Supplementary Figures S4a–c).

In the raw leachate, 107 MGE-linked contigs were identified, associated with 50, 23, and 18 bacterial species during sampling in autumn, spring, and summer, respectively. Key hosts included *Streptococcus*, *Staphylococcus*, and *Halomonas*, each harboring multiple MGEs (*N* ≥ 3). The primary MGEs identified were *tnpA* (transposase) and *IS91* (insertion sequence). Following ultrafiltration treatment, MGE-linked contigs increased to 247, with major hosts shifting to *Pseudomonas_E* and *Stutzerimonas*, both carrying diverse MGEs (*N* ≥ 3). Similarly, the number of bacterial species hosting MGEs rose to 119, 58, and 30 for the respective sampling periods. The dominant MGEs, however, remained *tnpA* and *IS91*. In the fully treated leachate, 294 MGE-linked contigs were identified, with *Pseudomonas_E* and *Acidovorax* as the primary hosts. The diversity of bacterial hosts further expanded to 106, 60, and 74 species across the sampling periods. These results indicate that leachate treatment, particularly ultrafiltration, does not reduce MGE-related bacterial loads but instead increases the diversity of bacterial hosts. This heightened host diversity amplifies the potential risk of ARG dissemination from leachate sources.

Furthermore, a preliminary analysis was conducted to explore the host distribution of MGEs within groundwater samples. At site BG1, a total of 350 contigs harboring MGEs were identified, corresponding to 262 distinct bacterial species. Notably, *Pseudomonas_E* and *Hydrogenophaga* were identified as the primary bacteria with MGEs (*N* ≥ 3). *TnpA* was found to be the MGE with the broadest host range at BG1. Intriguingly, the diversity of MGE hosts at BG1 varied with the sampling season. Specifically, in autumn, spring, and summer, the number of bacterial species carrying MGEs was 128, 90, and 82, respectively. At site BG3, 225 contigs with MGEs were identified, representing 182 different bacterial species. *Acidovorax* and *Pseudomonas_E* were the primary carriers of MGEs at this site (*N* ≥ 3). *TnpA* also exhibited the highest number of bacterial hosts in the BG3 samples. Seasonal analysis revealed 110, 19, and 62 bacterial species with MGEs in autumn, spring, and summer, respectively. For site BG5, 187 contigs containing MGEs were identified, associated with 153 bacterial species. *Azonexus* and *Pseudomonas_E* were the main bacterial hosts for MGEs at BG5 (*N* ≥ 3). *TnpA* maintained the highest number of bacterial hosts in the BG5 samples, with 39, 24, and 94 bacterial species carrying MGEs in autumn, spring, and summer, respectively.

### 3.12 Dual track risk ranking of ARG subtypes

**Figure 7:**
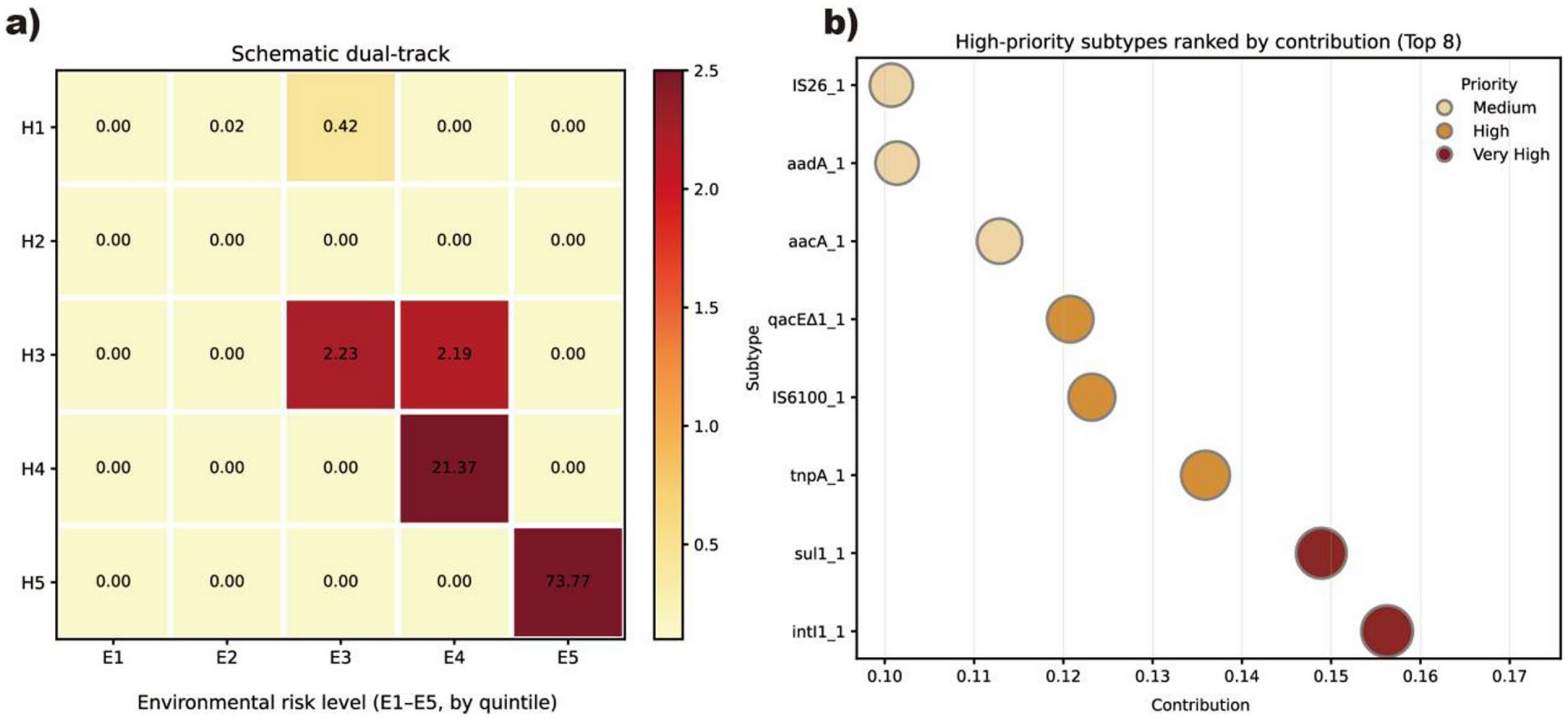
Dual-track risk assessment results of antibiotic resistance genes. **(a)** An overview of the ARG risk assessment results; **(b)** shows the risk levels of major high-risk ARGs.

Using this framework, we ranked all ARG subtypes included in the dual-track evaluation. Each subtype was assigned one health risk level (H1–H5) and one environmental risk level (E1-E5). Subtypes were then placed into the 5 × 5 dual-track matrix, and we summarized the cell-wise burden by summing the mean abundance (Total Abundance) of subtypes within each cell and normalizing it to the total (Figure 7a). The heatmap shows a highly skewed distribution: most cells contributed negligibly and appeared pale, whereas pronounced intensity was restricted to the high–high region. Specifically, H5-E5 alone accounted for 73.77% of the total abundance-weighted burden, and H4-E4 accounted for 21.37%; together these two cells comprised >95% of the total. Minor contributions were observed in H3–E3 (2.23%) and H3-E4 (2.19%), while all remaining cells collectively contributed <1%. Thus, within this dataset, the abundance-weighted burden was concentrated in a small fraction of subtypes that combined high health relevance with high environmental persistence/occurrence, rather than being evenly distributed across the matrix.

Consistent with this pattern, the top contributors were dominated by a small set of highly prevalent, high-priority subtypes (Figure 7b). The Top 8 subtypes ranked by contribution were *intI1, sul1, tnpA, IS6100, qacE, aacA, aadA, and IS26*, which together contributed 22.1% of the total abundance across all detected subtypes. Notably, several of these markers (*intI1, tnpA, IS6100, IS26, and qacEΔ1*) reflect integron/transposon/insertion-sequence-associated mobility and co-selection potential, while sul1 and aminoglycoside resistance genes (*aacA, aadA*) represent clinically relevant resistance functions. Their dominant contributions and concentration in the high–high region suggest that monitoring and engineering control should prioritize this compact set of resistance and mobility determinants to mitigate dissemination along the landfill leachate-groundwater continuum. To align with mainstream resistance-mechanism taxonomies, these Top-8 determinants can be grouped into (i) mobility/HGT markers (*intI1*, *tnpA*, *IS6100*, *IS26*), (ii) co-selection/biocide-associated efflux marker (*qacEΔ1*), (iii) metabolic bypass/target replacement (*sul1*), and (iv) enzymatic inactivation via aminoglycoside-modifying enzymes (*aacA*, *aadA*). Within the Top-8 contribution pool (22.1%), these four classes account for 51.6, 12.1, 14.9, and 21.4%, respectively, because H/E thresholds and leakage weights are dataset-derived, the dual-track scores should be interpreted as within-system prioritization rather than externally calibrated risk estimates, pending multi-site validation.

Otherwise, a joint analysis of the dual track risk matrix and the MGE abundance heatmap shows that ARG subtypes with high health and high environmental risk are rarely isolated. Instead, they tend to occur in samples where MGEs are also highly enriched. The high-high region was primarily driven by mobility- and co-selection–linked markers (*intI1*, *tnpA*, *IS6100*, *IS26*, *qacEΔ1*) together with clinically relevant resistance genes (*sul1*, *aacA*, *aadA*), consistent with the Top-8 contribution ranking in Figure 7b. This co distribution pattern suggests that high H and high E ARGs combine strong clinical relevance and pronounced environmental amplification with active embedding in a horizontally mobile MGE network. During their migration from landfill leachate to groundwater, they are therefore not only passively transported with water but also able to spread between hosts via plasmids and integrons, which makes them core risk units that should be prioritized in process safety management and engineering interventions.

## 4 Discussion

### 4.1 Inadequacy of conventional treatment: a process safety perspective

While the existing leachate treatment process effectively reduced conventional pollutants (e.g., COD, heavy metals) consistently across seasons—demonstrating reliability in managing traditional parameters—it exhibited significant limitations in mitigating ARGs and MGEs (Li F. et al., 2024; Le Page et al., 2017; Li H. et al., 2024). This deficiency underscores a critical engineering gap in biowaste treatment: the failure to address emerging biological risks, potentially leading to cross-media contamination where pollution is transferred from solid waste (leachate) to water resources (groundwater) rather than achieving resource-oriented mitigation (Li et al., 2025). The increase in ARG diversity and abundance post-treatment, particularly after ultrafiltration, highlights that current engineering designs may inadvertently amplify resistance risks by reshaping the microbial ecology—favoring resistant bacteria like *Pseudomonadota* (e.g., *Pseudomonas_E*) instead of aligning with sustainable biowaste processing goals (Lucidi et al., 2024; Liu et al., 2021; Luo et al., 2024). These findings emphasize that pollution control should prioritize overall risk reduction over mere contaminant transfer. Host assignments for MGE-carrying contigs are based on short metagenomic assemblies and should be interpreted as putative rather than definitive linkages.

### 4.2 Seasonal dynamics and engineering reliability

Seasonal variation was a major driver of ARG profiles, with higher abundance and diversity in autumn and summer (among the sampled seasons). Importantly, these seasonal peaks coincided with shifts in dominant ARG-host communities and with increased prevalence of mobility determinants (MGEs) and their host breadth, which is consistent with enhanced persistence and mobility of resistance determinants and thus a higher potential for downstream groundwater impact (Nalladiyil et al., 2025; Sharma et al., 2023). For instance, the proliferation of Pseudomonas (ARG hosts) in warmer months suggests biological treatment units operate suboptimally outside narrow climatic ranges, compromising year-round resource protection performance (Shen et al., 2023; Sun et al., 2021). Such season-dependent efficiency underscores the need for climate-adaptive process designs—e.g., adjustable hydraulic retention time (HRT) or dissolved oxygen (DO) control—tailored to semi-arid regional swings, ensuring consistent risk mitigation regardless of temporal changes. This highlights the importance of dynamic risk assessment for biowaste treatment products under uncertain climatic conditions.

### 4.3 Horizontal gene transfer risks and MGE proliferation

The strong resistome similarity between treated leachate and groundwater (revealed by PCoA and SourceTracker) confirms ARGs are not confined to the treatment system but migrate to downgradient aquifers. Beyond hydraulic transport, abundant MGEs enable ARG HGT, creating a more persistent hazard that conventional treatment fails to address.

The pronounced enrichment of mobility determinants in the system—particularly *intI1, tnpA*, and insertion-sequence markers (*IS6100 and IS26*)—suggests elevated HGT potential in treated leachate and downstream groundwater. Beyond overall MGE enrichment, the occurrence patterns of the mobilome in this semi-arid leachate-groundwater system were characterized by (i) strong dominance of a few core subtypes, (ii) a “richness-abundance decoupling” during treatment, and (iii) a spatial gradient in groundwater consistent with leachate influence. First, across all matrices and seasons, the mobilome was consistently dominated by transposition- and insertion-sequence-related determinants, with tnpA and IS91 remaining among the most prevalent subtypes, while plasmid-associated signals were comparatively low. Second, the treatment train tended to reduce the detection rate (i.e., the fraction of MGE subtypes detected) but markedly increased the abundance of dominant subtypes, indicating that treatment may remove rare/low-abundance MGE signatures while simultaneously enriching core mobility machinery. For example, in autumn the detection rate decreased from 84% (IW1) to 67% (EW1), yet *tnpA*, *IS91*, and *istA* increased from 152.64 → 1265.75, 32.88 → 248.68, and 16.59 → 64.13 in average relative abundance, respectively. In spring, tnpA showed an even stronger surge (245.79 → 4238.42, 17.4-fold), whereas IS91 and intI1 decreased, suggesting that different MGE classes responded heterogeneously to seasonal operation and selective pressures. In summer, the detection rate increased sharply (68% → 95%), together with increased *IS91* and *tnpA*, implying higher microbial activity and/or broader mobilome activation under warmer conditions. Third, groundwater samples shared the same core dominant MGEs as leachate and exhibited a spatial gradient (e.g., higher average *tnpA* at BG1 than BG5), consistent with downgradient influence from the landfill/leachate system. Collectively, these occurrence features support an engineering interpretation that the treatment system may act as a selective filter that concentrates key mobility determinants (rather than uniformly reducing mobilome signals), thereby elevating the potential for ARG persistence and horizontal dissemination in the receiving groundwater. Consistent with this mobility-enriched background, the dual-track risk matrix further indicated that the abundance-weighted burden was highly concentrated in the high-high region (Figure 7a), and the Top 8 subtypes ranked by contribution were dominated by integron/transposon-associated markers (*intI1, tnpA, IS6100, IS26*) together with co-selection and resistance genes (*qacEΔ1, sul1, aacA, aadA*) (Figure 7b). Among these, *intI1* and *sul1* are particularly critical because class 1 integrons can capture and disseminate resistance gene cassettes, making them practical priority targets for monitoring and technology optimization.

From a biowaste treatment optimization perspective, this combination of hydraulic connectivity, MGE proliferation, and mobile high-priority ARGs undermines the treatment system’s role as a resource protection barrier. It also justifies using our engineering-oriented dual-track risk matrix to prioritize ARG control. Below, we apply this matrix to identify high-risk ARGs, and in Section 4.5, translate these into concrete treatment optimization strategies.

### 4.4 Groundwater contamination: evidence of pollution migration

PCoA and linear regression revealed strong resistome similarity between treated leachate and adjacent groundwater—especially at landfill-proximal sites (e.g., BG1). These patterns indicate ineffective pollution containment and existing leakage pathways, consistent with recent reports (Tian et al., 2023; Wang et al., 2020). SourceTracker analysis further confirmed treated leachate as the dominant source of groundwater ARGs, demonstrating failure of resource protection barriers. In Hohhot—a semi-arid region where groundwater is a critical recycled water source—this transfer poses direct threats to water resource security and public health.

In the dual-track assessment, only a small subset of subtypes dominated the overall burden: the Top 8 high-priority subtypes—*intI1, sul1, tnpA, IS6100, qacEΔ1, aacA, aadA, and IS26*—together accounted for 22.1% of the total abundance-weighted contribution, highlighting a compact set of resistance- and mobility-linked determinants as core targets for resource-oriented monitoring and engineering control.

### 4.5 Engineering implications, risk control strategies, and research needs

Our results confirm the current landfill leachate treatment system acts as an incomplete barrier against antibiotic resistance. While conventional pollutants (COD, nutrients, heavy metals) are efficiently removed, high-risk ARGs and MGEs persist—or even accumulate—in treated effluent and groundwater. Seasonal fluctuations further reduce control stability, with the system amplifying ARGs/MGEs/resistant hosts in warm periods instead of suppressing them. This behavior reveals a critical biowaste treatment flaw: the treatment train redistributes resistance risks rather than eliminating them (Wu et al., 2023; Wang Y. et al., 2022; Wu et al., 2024; Yang et al., 2024). This subtype/type aggregation does not fully resolve allele-level variation or distinguish chromosomal versus plasmid contexts for highly similar ARGs, which should be addressed by context-resolved analyses in future work.

To address this, targeted technology upgrades and resource-protective measures are required: (1) Process intensification for ARGs/MGEs co-removal: Upgrade treatment trains with advanced oxidation + nanofiltration hybrid systems to damage extracellular DNA, inactivate resistant hosts, and reduce MGEs—instead of focusing solely on bulk organic/nutrient removal; (2) Biological risk-informed monitoring: Incorporate biological indicators into routine discharge criteria. Track key ARG subtypes (via real-time qPCR), MGEs activity, and high-risk hosts (Pseudomonas_E) to enable quantitative resistance risk management. (3) Seasonal adaptive operation: Adjust HRT, sludge retention time (SRT), and disinfection intensity to maintain performance in warm, high-risk seasons—aligning with climate-resilient biowaste treatment goals; (4) Physical barrier reinforcement: Design groundwater monitoring wells, impermeable liners, and leakage detection systems as part of inherent resource protection to block leachate-groundwater transfer.

### 4.6 Limitations and future perspectives

This study elucidates the distribution and removal characteristics of ARGs and MGEs in the landfill-leachate-groundwater continuum of semi-arid regions, and clarifies the ARGs abatement performance of core membrane treatment units, yet several inherent limitations merit acknowledgment. First, the NF sampling port of the studied leachate treatment plant was closed for routine maintenance during sampling, precluding the collection of NF permeate and concentrate. This restricts fine-grained attribution of ARGs removal efficiency to individual membrane units (NF vs. reverse osmosis, RO), such that ARG profile differences between UF permeate and RO effluent can only be interpreted as the combined effects of NF and RO rather than standalone unit performance. Second, winter sampling was not conducted due to extreme low temperatures, frozen sampling infrastructure and site access restrictions in the semi-arid study area; given the long, cold winters in such regions are characterized by drastically reduced microbial activity and distinct groundwater recharge dynamics, the current results only reflect ARGs seasonal variations across spring, summer and autumn, failing to capture complete annual ARG dynamics and leading to an incomplete analysis of ARGs environmental responses. Third, groundwater flow paths were not modeled in this study, and the dual-track matrix—developed for a single semi-arid system-serves only as a qualitative screening tool for engineering prioritization rather than a fully calibrated quantitative risk model. It lacks explicit resolution of dose–response relationships and site-specific exposure scenarios, relies on external public databases for the health risk track, and its H1-H5/E1-E5 scoring system has not undergone external validation, limiting its robustness and generalizability across landfill types, treatment trains and climatic contexts.

These limitations further identify clear priorities for future research and technology development. First, follow-up studies will coordinate with plant operation schedules to collect NF permeate and concentrate, quantifying the independent ARG removal efficiency of NF and RO to achieve accurate characterization of the membrane treatment process chain. Optimized low-temperature sampling protocols – including insulation/heating modification of sampling pipelines, cold-resistant sampling equipment and strict low-temperature sample preservation – will also be developed to implement year-round monitoring covering winter, enabling characterization of complete annual ARGs/MGEs seasonal dynamics and identification of key environmental drivers of ARGs fate in cold periods. Second, metagenomic risk indicators will be integrated with hydrogeological-omics coupling models to quantify transport distances and time scales of high-priority ARGs. The dual-track matrix will be further refined via external validation through multi-site application across varying landfill ages and hydroclimatic regimes, benchmarking against qPCR-derived absolute ARGs loads, and sensitivity analyses of leakage weights and quintile thresholds to quantify uncertainty; hydrogeological transport modeling will be coupled with this framework to further constrain high-priority ARG transport dynamics. Recalibration of the dual-track matrix for other landfill types and climatic zones will also be performed to enhance its transferability and quantitative risk assessment capacity.

Notwithstanding these limitations, the dual-track approach developed in this study effectively identifies locally critical ARGs, links metagenomic data to biowaste treatment optimization, and provides a practical guidance framework for safer leachate management in water-scarce semi-arid regions (Zou et al., 2020). Such insights lay a foundation for the targeted control of ARGs/MGEs risks in landfill-leachate-groundwater systems, with future refinements set to further enhance its engineering value and generalizability.

## 5 Conclusion

This study demonstrates that the current landfill leachate treatment system acts as an incomplete barrier for antibiotic resistance control in biowaste treatment. Conventional pollutants are efficiently removed, yet ARGs and MGEs persist—or even accumulate – in treated effluent and adjacent groundwater, posing risks to groundwater resource security (Wang L. et al., 2022; Zhou et al., 2025). Seasonal variability further undermines control efficacy, favoring the enrichment of resistance determinants in warm periods. By integrating resistome profiling, mobilome characterization, and an engineering-oriented dual-track health–environmental risk framework, we found that the abundance-weighted burden was concentrated in a compact set of resistance- and mobility-linked determinants. The top-8 contributors—*intI1*, *sul1*, *tnpA*, *IS6100*, *qacEΔ1*, *aacA*, *aadA*, and *IS26*—accounted for 22.1% of the total abundance-weighted contribution, and were dominated by integron/transposon/insertion-sequence markers together with clinically relevant resistance genes, highlighting practical targets for monitoring and treatment optimization (Yu et al., 2016).

These findings imply that landfill leachate management should explicitly integrate ARGs/MGEs-targeted control into leachate treatment process optimization, adopt seasonally adaptive operation strategies, and incorporate key resistance indicators into routine engineering monitoring (Berendonk et al., 2015; Jin et al., 2022). The dual-track risk matrix developed here serves as an engineering screening tool to identify locally critical ARGs and guide safer leachate management in data-scarce semi-arid regions (Klümper et al., 2025). Meanwhile, the underlying thresholds and leakage weights are site-specific and will require recalibration and validation for broader biowaste treatment scenarios when applied to other systems.

## Data Availability

The datasets presented in this study can be found in online repositories. The names of the repository/repositories and accession number(s) can be found at: http://www.ncbi.nlm.nih.gov/bioproject/1306755, 1306755 and http://www.ncbi.nlm.nih.gov/bioproject/1306772, 1306772.

## Footnotes

1
http://www.usadellab.org/cms/uploads/supplementary/Trimmomatic

2
http://bio-bwa.sourceforge.net/bwa.shtml

3
http://www.cloud.biomicroclass.com/CloudPlatform
