# Supplementary material for: Control efficacy and groundwater risk of antibiotic resistance genes in semi-arid landfill leachate treatment: seasonal insights and engineering implications
Source: Front Microbiol. 2026 May 15;17:1807935. doi: 10.3389/fmicb.2026.1807935 (PMC13219371; doi:10.3389/fmicb.2026.1807935)
Supplement: Supplementary file 2 [file Data_Sheet_2.DOCX]

**Supplementary Material**

**Control effect of antibiotic resistance genes and associated groundwater risk in landfill leachate treatment systems of semi-arid regions: seasonal variations and engineering implications**

**Ning Chang^1†^, Nan Li^2†^, Wenhao Li^1^, Jiaying Xue^1^, Yuhong Zheng^3^, Chengzhen Zhao^4^, Shenghu Zhang^1,5,6^, Guangxuan Yin^1^, Miaoyi Bao^1*^, Weitao Shen^1*^**

^1^Department of Engineering, China Pharmaceutical University, Nanjing 211198, China

^2^Department of Gastroenterology, Zhongda Hospital affiliated to Southeast University, Nanjing, 210009, China

^3^Department of Environment Health, Nanjing Municipal Center for Disease Control and Prevention, Nanjing 210003, China

^4^Institute of Agricultural Resources and Environment, Yunnan Academy of Agricultural Sciences, Kunming 650205, China

^5^Nanjing Institute of Environmental Sciences, Ministry of Ecology and Environment, Nanjing 210042, China

^6^Eastern Regional Technology Center of Hazardous Waste Environmental Risk Prevention and Control, Nanjing 210032, China

*** Correspondence:**

*Miaoyi Bao: Department of Engineering, China Pharmaceutical University, Nanjing 211198, China. E-mail address:baomiaoyi@163.com

*Weitao Shen: Department of Engineering, China Pharmaceutical University, Nanjing 211198, China. E-mail address: [1520240185@cpu.edu.cn](mailto:1520240185@cpu.edu.cn)

^†^ Ning Chang and Nan Li contributed equally to this work.

**Table. S1. Basic physicochemical properties, heavy metals, and antibiotic contents in all samples**

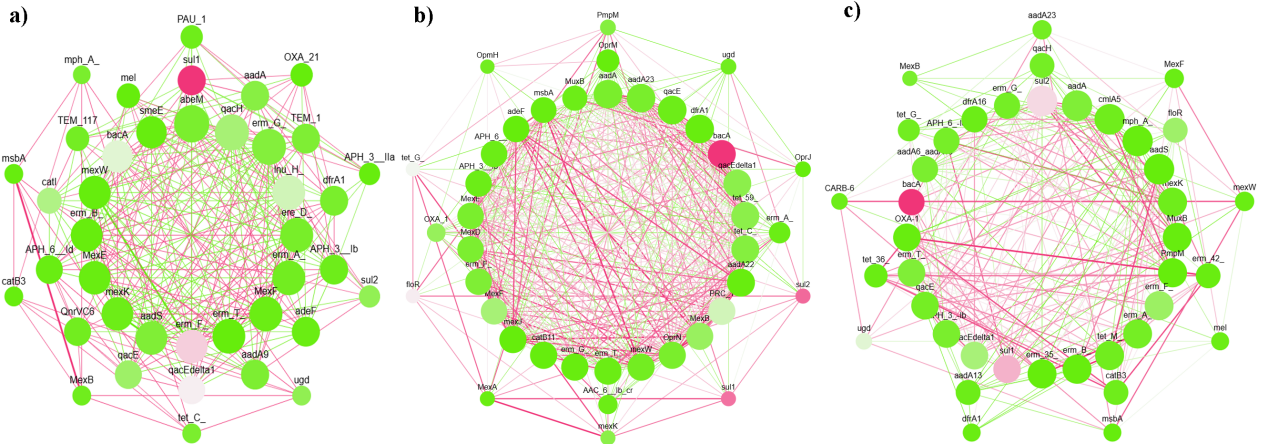


**Fig. S1 a)-c) represent the co-occurrence networks of ARGs during the leachate treatment process in autumn, spring, and summer, respectively.**

**
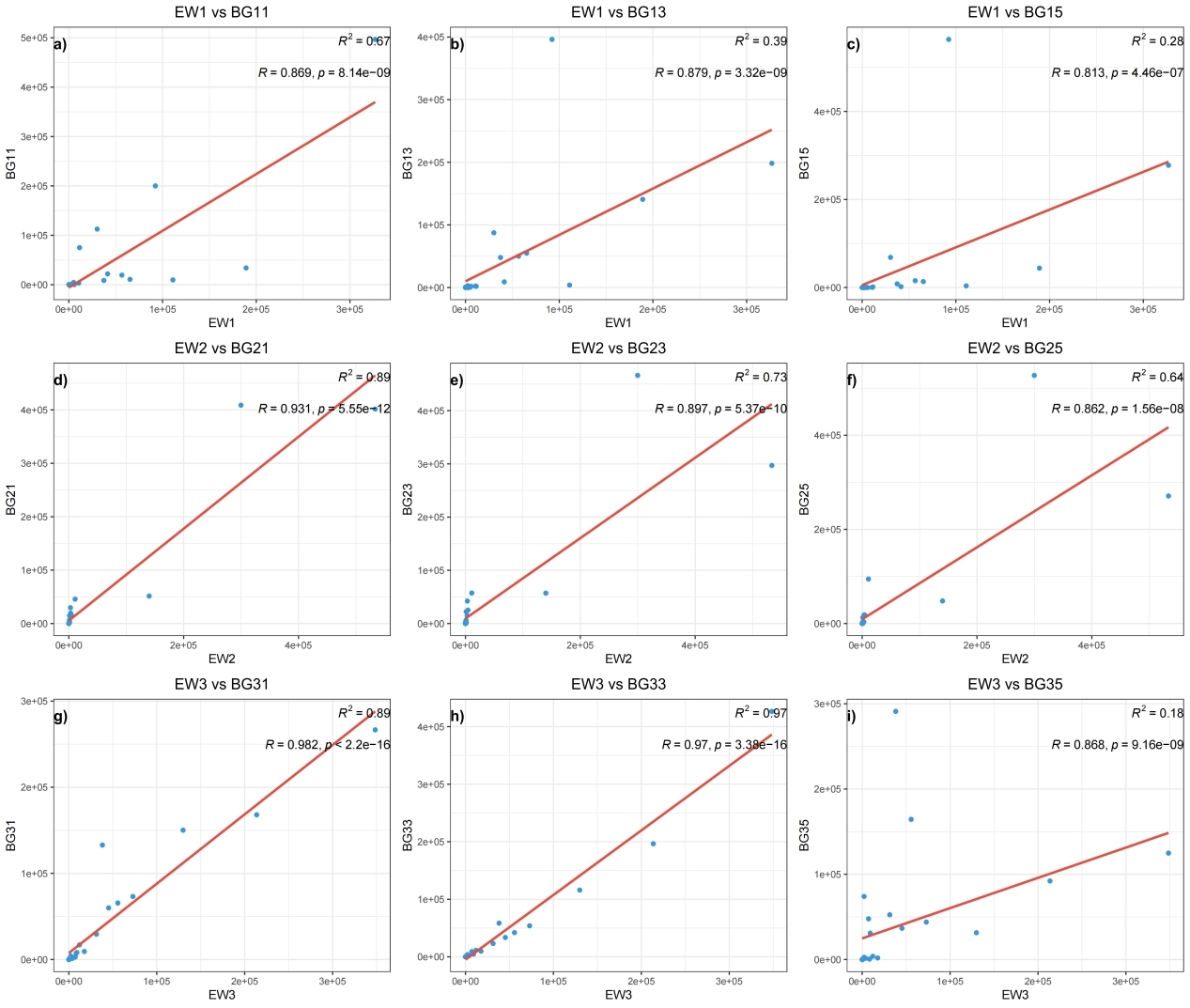
**

**Fig. S2 Linear correlation analysis of antibiotic resistance gene (ARG) abundance in treated leachate and groundwater.**

**
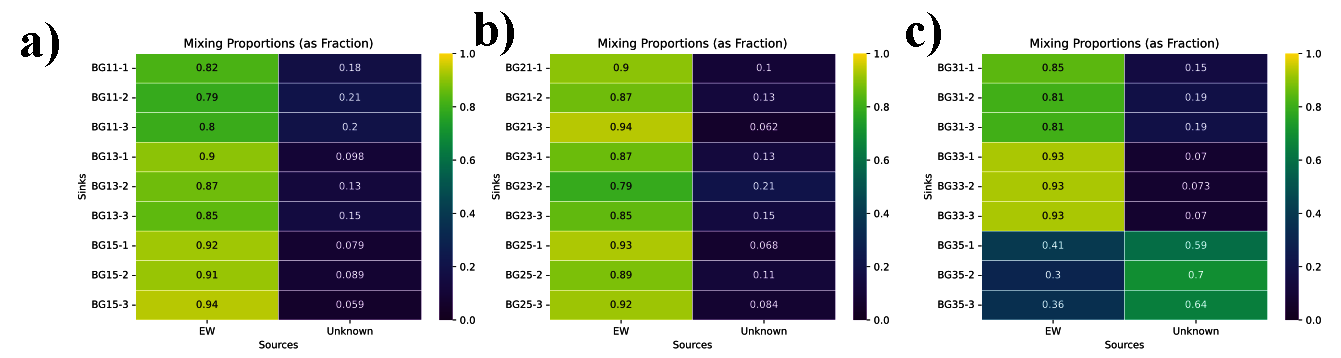
**

**Fig.S3 Source tracing analysis of leachate after treatment of antibiotic resistance genes in groundwater based on Sourcetracker**


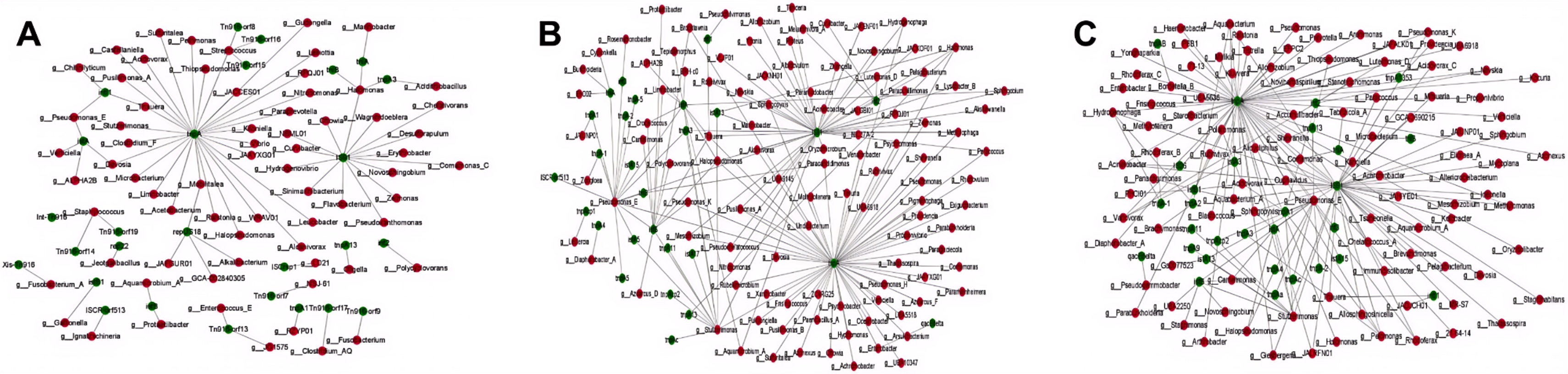


**Fig. S4 (A) stands for host of MGEs in raw leachate. (B) stands for host of MGEs in ultrafiltration leachate. (C) stands for host of MGEs in treated leachate.**
